# In-silico study of rosmarinic acid roles in inhibiting breast cancer progression

**DOI:** 10.37796/2211-8039.1638

**Published:** 2025-03-01

**Authors:** Ngakan Putu Krishna Mahayana, Ngurah Bagus Agung Surya Nanda Jayesvara Dwi Sutanegara, Made Dwinanda Prabawa Mahardana, Desak Made Wihandani

**Affiliations:** a School of Medicine, Faculty of Medicine, Udayana University, Denpasar, Bali, Indonesia; b Department of Biochemistry, Faculty of Medicine, Udayana University, Jl. PB. Sudirman, Denpasar, 80232, Bali, Indonesia

**Keywords:** Breast cancer, In-silico, Rosmarinic acid

## Abstract

**Background:**

Breast cancer is the highest cancer incidence in the world. Chemotherapy is currently one of the main breast cancer treatments besides surgery. It is capable of evolving to become resistant to chemotherapy agents. Chemotherapy also has significant side effects. Rosmarinic acid could become an anti-cancer agent candidate for the treatment of breast cancer, but its molecular mechanism is still unclear.

**Aim:**

This study aimed to clarify the molecular mechanism of rosmarinic acid anti-breast cancer properties via an in-silico study.

**Methods:**

Web-based screening tools such as SwissTargetPrediction, Similarity Ensemble Approach (SEA), and TargetNet were used as initial screening. From web-based screening, potential proteins that interact with rosmarinic acid could be determined. Intersected proteins from 3 web-based screenings were assessed via literature review. We found 11 intersected proteins, and 6 of 11 proteins are involved in breast cancer development and progression. Those 6 proteins are MMP-1, MMP-2, MMP-9, MMP-12, aldose reductase, and M-phase Inducer Phosphatase 2 (CDC25B). Then molecular docking using Autodock 4.6.2 was used in ligand and protein interaction simulation. Those 6 proteins were selected as macromolecules in the docking study.

**Results:**

Based on the docking result, we found that rosmarinic acid can bind MMP-1, MMP2, MMP-9, and MMP-12 active sites. The binding profile of rosmarinic acid with aldose reductase has similarities with other confirmed inhibitors. Docking with CDC25B showed that rosmarinic acid also binds in the same place as cyclin-dependent kinases (CDKs).

**Conclusion:**

The ability of rosmarinic acid to inhibit MMP-1, MMP-2, MMP-9, aldose reductase, and CDC25B activity may underlie how rosmarinic acid is able to inhibit the development of breast cancer.

## 1. Introduction

Based on GLOBOCAN 2018 data, there are 2.3 million breast cancer patients in the world [1]. Breast cancer is also cancer with the highest incidence among other types of cancer [2]. Chemotherapy is currently one of the main breast cancer treatments besides surgery [3]. The use of chemotherapy has two weaknesses: cancer could evolve and become resistant to several chemotherapy agents and chemotherapy also has significant side effects due to lack of selectivity. Based on that reason, the development of novel anti-cancer agents is required [4].

Chemotherapy led to a significant incidence of severe adverse effects, impacting nearly 44.5% of treated patients [5]. Chemotherapy lack of specificity leads to toxic effects in previously healthy organs, most commonly the heart, liver, kidneys, bone marrow, and nervous system. These toxicities can be either acute or chronic, persisting even after treatment concludes which could affect pain management and discharge planning [6]. The increasing resistance to chemotherapeutic agents such as doxorubicin, paclitaxel, 5-fluorouracil [Vulsteke], cyclophosphamide, and carboplatin are linked to a growing incidence of breast cancer recurrence and worse outcomes in terms of prognosis and survival [7–11]. Approximately 80–90% of cancer-related deaths are linked to drug resistance [12]. Therefore, the development of novel anti-cancer agents is required especially for breast cancer.

**Fig. 1:**
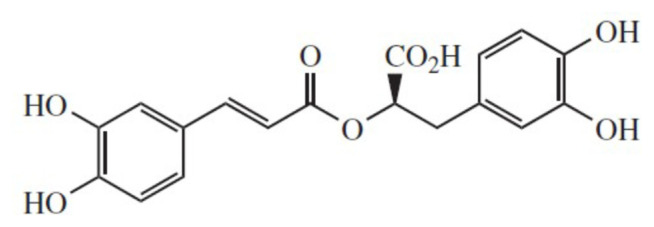
Rosmarinic acid structure.

Rosmarinic acid can be found in several plants, especially family *Lamiaceae, Araliaceae, Cucurbitaceae, Rubiaceae*, and *Plantaginaceae* [13]. This compound can be classified as a secondary phenolic molecule. The rosmarinic acid structure is presented in Fig. 1 [14]. Rosmarinic acid is known for its ability as an anti-cancer agent. Several research have found that Rosmarinic acid can inhibit cancer proliferation including breast cancer [15–18]. A recent study by Mahmoud *et al*. [16] discovers that Rosmarinic acid can suppress inflammation, angiogenesis, and improve paclitaxel-induced apoptosis in a breast cancer model via modulating the NF3 κb-p53-caspase-3 pathways. However, this finding still couldn't completely confirm the underlying pharmacodynamic properties of rosmarinic acid in inhibiting breast cancer progression. Previous studies also suffer from the same problem. Understanding the link between cancer, apoptosis, and drug resistance pharmacodynamics is crucial for developing effective molecular-targeted therapies. Therefore, this study aims to clarify the molecular mechanism of Rosmarinic acid in the inhibition of breast cancer progression [19].

## 2. Aim

This research aimed to find the molecular mechanism of Rosmarinic acid in inhibiting breast cancer proliferation with in-silico methods.

## 3. Methods

### 3.1. Protein target screening

Web-based in-silico screening tools were used in the initial screening for protein target determination. Following web-based tools were used: Swiss Target Prediction (http://www.swisstargetprediction.ch/) (SIB Swiss Institute of Bioinformatics, Lausanne, Switzerland) [20], Similarity Ensemble Approach (SEA) (https://sea.bkslab.org/) (Shoichet Lab., UCSF, San Francisco, California, USA) [21], and TargetNet (http://targetnet.scbdd.com) (SCBDD, Changsha, Hunan, China) [22]. Proteins selected from SwissTargetPrediction, TargetNet, and SEA were inserted into the Venn diagram using an automated web-based tool called InteractiVenn (http://www.interactivenn.net/) (Instituto de Ciências Matemáticas e de Computação, São Carlos, Brazil) to determine the protein that intersected in the previous three databases [23]. The proteins that are involved in breast cancer progression were selected for molecular docking.

### 3.2. Ligand preparation

Rosmarinic acid as a structure was downloaded from the PubChem website (https://pubchem.ncbi.nlm.nih.gov/) (National Library of Medicine, Bethesda, Maryland, USA) in SDF format [24]. Then ligand structure was optimized using Avogadro 1.2.0 [25].

### 3.3. Protein preparation

Protein's (.pdb) structures were downloaded from the Protein Data Bank website (https://www.rcsb.org/) (RCSB, San Diego, California, USA) [26]. Proteins were cleaned using Biovia Discovery Studio Visualizer 21.1.0 (Dassault Systèmes, Paris, France) then protein energy was minimized using Swiss-PDB Viewer 4.1 (SIB Swiss Institute of Bioinformatics, Lausanne, Switzerland) [27]. Polar hydrogen and Kolmann charge were added to the proteins using Autodock Tools 1.5.6 [28]. The resolution of the proteins must be lower than 3 Amstrong.

### 3.4. Molecular docking and visualization

Molecular docking was performed using Autodock tools 1.5.6 with Autodock 4.2.6 program. The Lamarckian and Genetic Algorithms were used in the docking process [28]. Docking results were downloaded as (.pdb) and visualized using Biovia Discovery Studio Visualizer 21.1.0. Docking validation was done by re-docking the native ligand from the crystallography structure with the protein. The docking process was considered to be valid if the Root Mean Square Difference (RMSD) of re-docking results is lower than 3 Amstrong [29]. The docking result is analyzed based on the docking profile. If there is no information about protein structure, docking results were compared to another confirmed ligand.

## 4. Result

### 4.1. Protein screening result

**Fig. 2:**
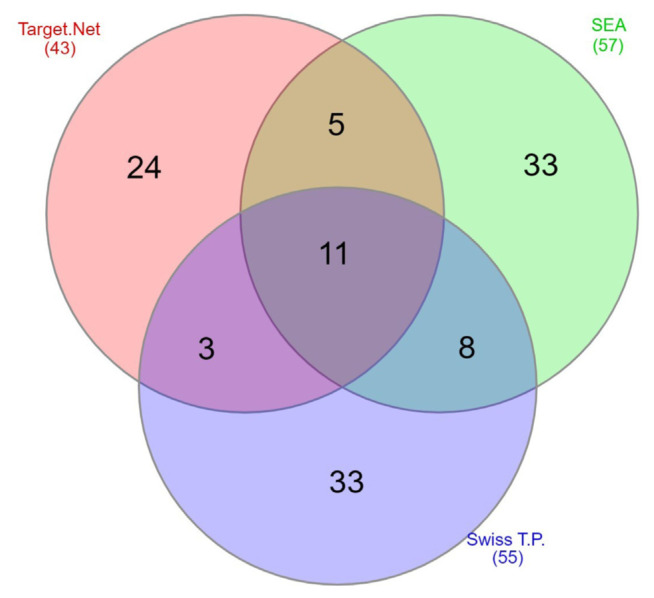
Protein screening venn diagram.

After the web-based screening method was performed, we found all human proteins that might interact with rosmarinic acid. Each web-based screening platform showed different results. Therefore, the screening results of each platform were inserted into venn-diagram to find intersected proteins. We found as many as 11 intersected protein candidates that might interact with rosmarinic acid. Those proteins are Aldose reductase, Carbonic anhydrase 4, Carbonic anhydrase 5A, Carbonic anhydrase 6, Carbonic anhydrase 7, Carbonic anhydrase 14, MMP-1 (Interstitial Collagenase), MMP-2, MMP-9, MMP-12, and M-Phase Inducer Phosphatase 2 (CDC25B). The venn-diagram of the result is shown in Fig. 2.

We performed literature review to select which protein candidates have roles in breast cancer progression. Among those 11 protein candidates, we found 6 proteins that are involved in the progression and development of breast cancer, those are MMP-1, MMP-2, MMP-9, MMP-12, M-phase inducer phosphatase-2 (CDC25B), and Aldose reductase [30–34]. Those 6 proteins were chosen as protein targets in molecular docking for rosmarinic acid.

### 4.2. Docking validation

Before performing the docking process between 6 chosen proteins and rosmarinic acid, we performed the re-docking process between those proteins with their known ligand that we acquired from available crystallography structure. All re-docking processes between the proteins and their native ligands have low RMSD values (RMSD <3 Amstrong). This finding suggests that the docking process in this study could imitate the results of *in vitro* or *in vivo* experiments [29]. The RMSD value of the redocking process and PDB code of all proteins are presented in Table 1.

### 4.3. Docking result

**Fig. 3:**
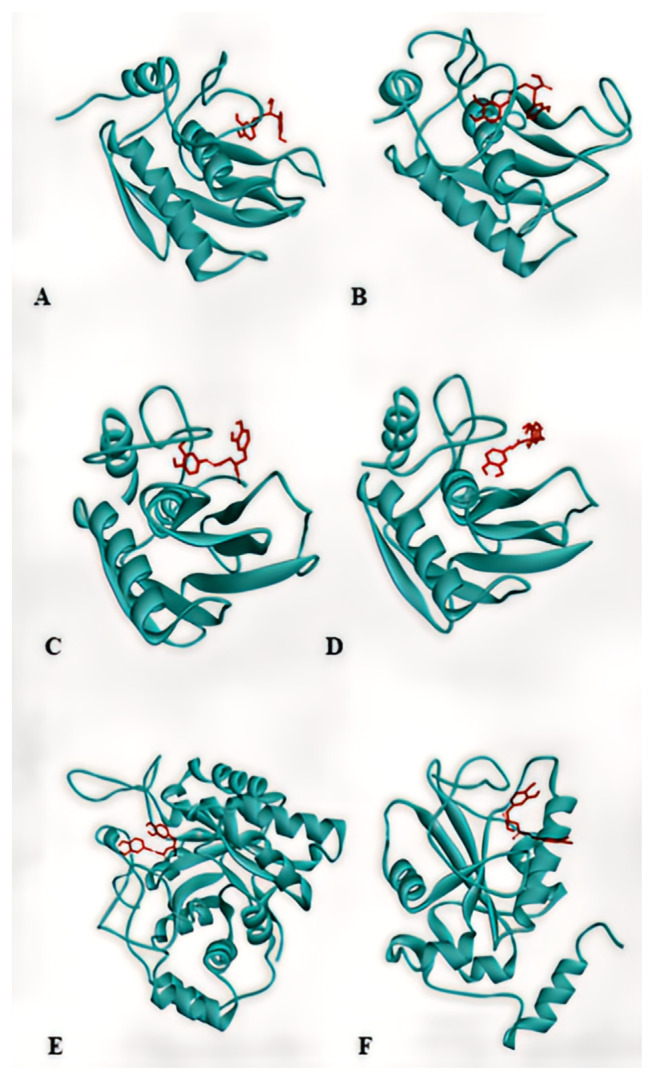
Docking results of rosmarinic acid with (A) MMP-1, (B) MMP-2, (C) MMP-9, (D) MMP-12, (E) aldose reductase, (F) CDC25B.

**Fig. 4:**
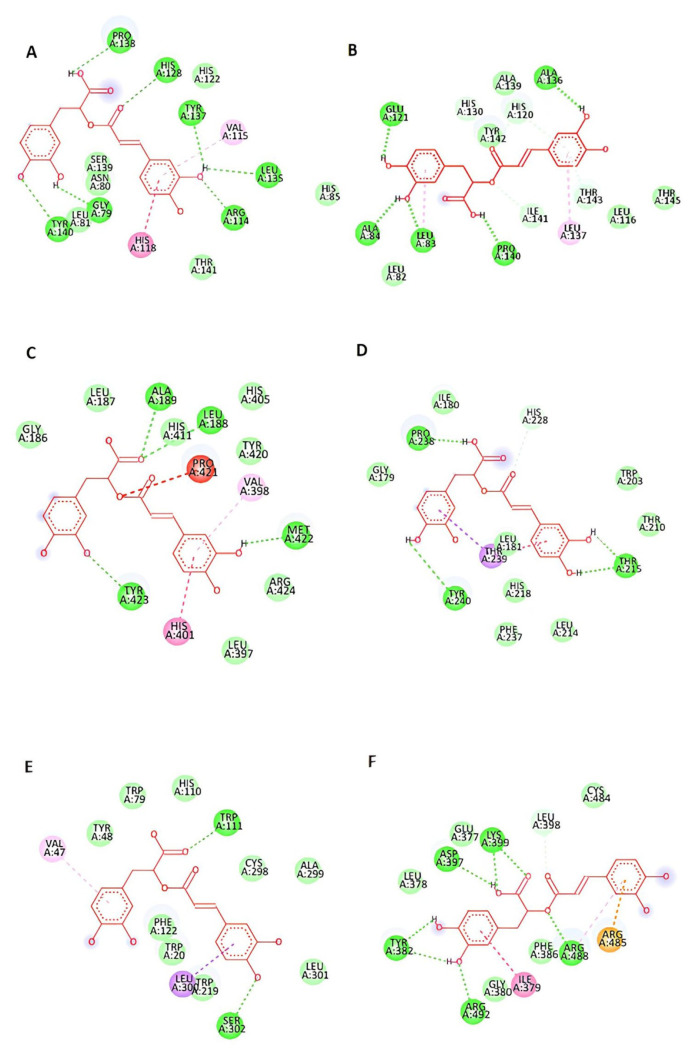
Docking profile of rosmarinic acid with (A) MMP-1, (B) MMP-2, (C) MMP-9, (D) MMP-12, (E) aldose reductase, (F) CDC25B.

**Fig. 5:**
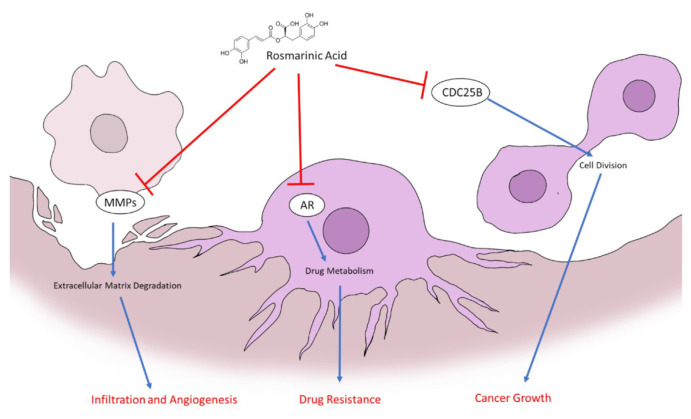
The ability of rosmarinic acid in inhibiting breast cancer progression.

Docking results of rosmarinic acid with 6 chosen proteins that are involved in breast cancer progression are shown in Fig. 3. The interactions of rosmarinic acid with all selected protein amino acids are shown in Fig. 4. Detailed information about the docking profile is presented in Table 2. We analyzed all docking results based on known information about proteins structure that we acquired from literature review. All docking results and their comparison to known information about protein structure is explained in following section bellow (Fig. 5):

#### 4.3.1. MMPs

The docking result between rosmarinic acid and MMP-1 showed that rosmarinic acid interacts with His118 and His128 on MMP-1, which are part of the consensus HexxHxxGxxH in MMPs [35,36]. The consensus HExxHxxGxxH is known as important part for MMPs catalytic activity [35,36]. Meanwhile, the docking result of rosmarinic acid with MMP-2, MMP-9, and MMP12 showed that rosmarinic acid binds with important amino acids on the catalytic site of those enzymes [37–40].

#### 4.3.2. Aldose reductase

This study also found that rosmarinic acid interacts with Trp111 and Leu 300 in aldose reductase via hydrogen bond. Trp111 is an important amino acid in the aldose reductase catalytic domain [41,42].

#### 4.3.3. CDC25B

The docking results of rosmarinic acid with M-Phase Inducer Phosphatase (CDC25B) found that rosmarinic acid interacts with Arg488 and Arg492. Both amino acids are the “hot spot” residue for CDC25B to recognize its protein substrate, Cdk2-CycA [43,44].

## 5. Discussion

Based on the results of this study, rosmarinic can bind with the consensus HExxHxxGxxH of the MMPs, which is known as part of the MMP-1 catalytic site [35,45,46]. The consensus HExxHxxGxxH consists of three histidines (His118, His122, and His128) that are responsible for the binding site of zinc ion [35,45,46]. Zinc ion is required as a co-factor for MMP's activity [46]. The crystallography of MMP-1 and its confirmed inhibitor also showed that its confirmed inhibitor binds with the consensus HExxHxxGxxH [35]. The interaction of rosmarinic acid with His118 and His128 could underlie its mechanism in inhibiting MMP-1 activity by preventing the interaction of zinc ions as the protein's co-factor.

Several studies have shown that MMP-1 can promotes cell proliferation of breast cancer and plays a role in angiogenesis and metastasis, which can further progress breast cancer [47–49]. According to certain studies, using inhibitors that bind to the catalytic site of MMP-1 could be a potential alternative therapy to halt the progression of cancer during its early stages [48,50]. However, this approach may not be effective for treating metastatic or advanced stages of cancer [48,50]. Based on our study result, rosmarinic acid binds to the catalytic site of MMP1. This finding suggests that rosmarinic acid may have potential as an alternative therapy for slowing the progression of early-stage breast cancer by inhibiting MMP-1.

Gelatinase-A or MMP-2 has been observed to be overexpressed in several types of cancer, including breast cancer [51]. The ability of MMP-2 to degrade the extracellular matrix of surrounding tissue enhances the migration of cancer cells, thus promoting the invasion of breast cancer cells [48,51]. Several studies found that overexpression of MMP-2 and MMP-9 correlated with breast cancer metastasis and poor prognosis [48,52,53]. Gelatinase-B or MMP9 can upregulate several genes as regulator of the malignant phenotype, also affecting the breast cancer phenotype [54]. The expression of MMP-9 mRNA has a positive correlation with other MMPs expression [53]. Several studies found that the inhibition of MMP-2 and MMP-9 can reduce breast cancer angiogenesis and metastasis [48,51,54–56]. However, several studies show that MMP-9 and MMP-12 not only have pro-tumor activity, but they also have anti-tumor activity due to their ability to inhibit angiogenesis *in vivo* [48,57]. In addition, MMP-12 A1082G gene polymorphism G allele has associated with poorer prognosis of breast cancer patients [32]. Some systematic reviews suggest that high expression of MMP-12 could be used as a biomarker for poorer prognosis due to shorter overall survival in breast cancer patient [48].

The docking results between rosmarinic acid and MMP-2, MMP-9, and MMP-12 showed that rosmarinic could bind at the active site of those proteins. These findings suggest that rosmarinic acid could be a competitive inhibitor that prevents the interaction of those proteins with their ligands [37–40]. The inhibitory potential of rosmarinic acid on MMP-1, MMP-2, MMP-9, and MMP-12 will limiting their ability to promote proliferation, invasiveness, angiogenesis, and metastasis of breast cancer cells [48,51,54–56,58]. Thus, potentially preventing further progression of the breast cancer.

In this study, we found that rosmarinic acid also binds with important amino acids in the aldose reductase catalytic site. This result is similar to the interaction with a known aldose reductase inhibitor, tolmetin [59]. Both rosmarinic acid and tolmetin interact with Trp111 and Leu300 in the aldose reductase [59]. This is in line with a previous in silico study that shows the Trp111 and Leu 300 are important amino acid residues that directly have an inhibitory effect for aldose reductase activity [42]. The ability of rosmarinic acid to inhibit aldose reductase was already found in the previous study by Kang *et al*. [60]. Thus, this study clarifies its property as a competitive aldose reductase inhibitor. Aldose reductase is a variant of the Aldo-Keto Reductase (AKR) class enzyme. AKR is involved in chemotherapy agent metabolism and eradicating stress condition that is given by chemotherapy agent [61]. AKR is also reported to be overexpressed in breast cancer [34]. An experimental model of aldose reductase inhibition showed promising results in treating chemo-resistance cancer including breast cancer [62]. Based on those findings, rosmarinic acid has a potential in treating chemoresistance breast cancer.

This *in silico* study also found that rosmarinic acid could bind with CDC25B in the same location as CDKs. This finding suggests that rosmarinic acid could prevent the interaction between CDC25B and CDKs. The interaction between CDC25B and CDKs is required for cell cycle progression [63]. A study by Ito *et al*. [64] reported that CDC25B overexpression occurs in breast carcinoma. Song *et al*. [65] found that the direct negative regulator of CDC25B inhibits breast cancer growth. Those findings and the current in silico study suggest that rosmarinic acid could prevent breast cancer proliferation by inhibiting cell cycle progression.

## 6. Conclusion

Based on the docking results, rosmarinic acid can inhibit MMP-1, MMP-2, MMP-9, and MMP12. Since those proteins are involved in breast cancer metastasis and angiogenesis, the ability to inhibit those proteins suggests that rosmarinic acid has the potential to inhibit breast cancer progression by preventing angiogenesis and metastasis. Rosmarinic acid can also inhibit aldose reductase. This finding suggests rosmarinic acid's potential in treating chemo-resistance breast cancer. Finally, rosmarinic acid's capability to inhibit the interaction between CDC25B and CDKs indicates its potential to inhibit cell cycle progression (Fig. 5).

## Figures and Tables

**Table 1:** Docking validation results.

Protein	PDB id	Re-docking RMSD	Crystallography Reference
MMP-1	2TCL	1.5	[28]
MMP-2	1HOV	2.37	[30]
MMP-9	1GKC	1.62	[31]
MMP-12	1RMZ	1.28	[32]
Aldose reductase	3S3G	2.9	[27]
CDC25B	4WH7	1.81	[30]

**Table 2:** Docking result of rormarinic acid. Bold is indicating important amino acid.

Protein	Binding coordinate	Amino acid residue interaction	Binding affinity (kcal/mol)
MMP-1	73.986, 8.655, 9.907	Arg114 Gly79 **His118** **His128** Leu135 Pro138 Tyr137 Tyr140 Val115	−6.26
MMP-2	5.367, 18.049, 21.566	**Ala83** **Ala84** His120 **Glu121** Pro140 Ile141 Ala136 Leu137 Thr143	−7.71
MMP-9	65.607, 31.083, 117.843	**Leu188** Ala189 Val398 His401 **Pro421** Met422 **Tyr423**	−6.47
MMP-12	6.478, 26.241, 2.993	His228 **Leu181** Pro238 Thr215 Thr239 Tyr240	−8.90
Aldose reductase	−9.484, 8.05, 21.05	Val 47 **Trp111** **Leu300** Ser 302	−5.71
CDC25B	16.84, −7.797, −4.002	Arg485 **Arg488** **Arg492** Asp397 Glu377 Ile379 Leu398 Lys399 Tyr382	−5.11
